# Dataset to develop self-report measure of emotional instability and behavioral difficulties for Malaysian youths

**DOI:** 10.1016/j.dib.2020.105795

**Published:** 2020-06-03

**Authors:** Nor Ba'yah Abdul Kadir, Nur Saadah Mohamad Aun, Norhayati Ibrahim, Hilwa Abdullah Mohd Nor, Diana Johan

**Affiliations:** Universiti Kebangsaan Malaysia

**Keywords:** Adolescents, Youths, Emotions, Behavior, Assessment, Malaysia

## Abstract

The article presents reliability statistics data in relation to the development of emotional instability and behavioral difficulties scale for youths in a Malaysia context. The data were obtained from youths participants in Kuala Lumpur and Klang Valley, Selangor, Malaysia. The data has four different subscales in describing emotional instability and behavioral difficulties. The data were analyzed using Cronbach's alpha, McDonald's ω, and Gutmann's λ6 to examine internal consistency test. The data showed that this new scale can be used to measure three subscales of emotional instability and one subscale of behavioral difficulties among youths in a Malaysia context.

Specifications table

**Table:** 

Subject	Psychology
Specific subject area	Developmental Psychology
Type of data	Table, Figure
How data were acquired	Field survey
Data format	Raw, analysed
Parameters for data collection	Sample comprises youths who are living in urban area of Kuala Lumpur and Klang Valley, Selangor, Malaysia
Description of data collection	Data of youths’ emotional instability and behavioural difficulties were collected from residential areas of urban poor. All participants who were present at the time of data collection and were able to take part to complete a set of questions.
Data source location	Kuala Lumpur and Klang Valley, Selangor, Malaysia
Data accessibility	Data are included in this article, http://dx.doi.org/10.17632/9ktv578p59.4

## Value of the data

• The data provides insights into culturally appropriate items in measuring emotional instability and behavioural difficulties among youths in a Malaysia context. The data can be used as a screening tool in developmental, social, clinical, and educational studies.
• The data also have value-added in providing a valuable contribution to develop a specific intervention program for youths to improve the youths’ emotional well-being and enhance their prosocial behaviour. The data also can be used "before" and "after" to audit everyday practice (eg., in rehabilitation centre or special schools) and to evaluate specific interventions (eg., parenting groups).
• Due to the nature of the data, further statistical analysis can be conducted including analysis of variance, factor analysis, and structural equation modelling. The data also can be used to conduct comparison in cross-cultural studies.

## 1 Data Description

Participants were a community sample of 429 at-risk youths (41.1% female; 57.4% male; 1.4% not identified) recruited from the low-cost housing in Kuala Lumpur and Klang Valley, Selangor, Malaysia. Youths who are approximately ages 13 to 24 years old (mean age 17.97 years old) were invited. In term of religion, the sample consists primarily of Muslims (86.9%) with the remaining participants identifying themselves as Budhha (6.3%), Christian (3.7%), Hindu (2.8%) and others (0.2%). No information is available for those participants who refused to complete some of the socio-demographic questions.

The data comprises nominal, interval and ordinal data and contains five demographic variables (sex, age, religion, educational attainment, employment) and four subscales of Emotional Instability and Behavioural Difficulties Scale (EIBDS): sadness, anger, fear, and hyperactivity-inattention. The Positive and Negative Affect Schedule (PANAS; 1) was used to measure emotion. This brief scale is comprised of 20 items, with 10 items measuring positive affect (e.g., excited, inspired) and 10 items measuring negative affect (e.g., upset, afraid). Each item is rated on a five-point Likert scale, ranging from 1 = very slightly or not at all to 5 = extremely, to measure the extent to which the affect has been experienced in the past week. Only the negative emotions were used to validate EIBDS constructs.

## 2 Experimental Design, Materials, and Methods

### 2.1 Scale development

The content of the self-report measure was drawn from a number of sources. From Goodman's work with the construct of emotional symptoms and behavioural problems [2], items reflecting sadness, fear, anger, and hyperactivity-inattention. The following features were also drawn from Achenbach's work [3], items reflecting behavioural problems. With the above considerations, the EIBDS was constructed, with the following goals in mind, to develop a valid and reliable self-report measure to quantify emotional instability and behavioural difficulties, to establish reference values for emotional instability and behavioural difficulties in general population, online community, at-risk and high-risk samples. The initial item pool for the EIBDS consists of 109 items designed to address emotional instability and behavioural problems among adolescents, including prosocial behaviour. A group of subject matter experts who are specialised in clinical psychology, counselling psychology, and developmental psychology was invited to check face validity and agreement of the items. The items were developed by examining extant measures of emotional instability and behavioural problems, focusing on the uniqueness, differences, and similarities pertaining to the Diagnostic and Statistical Manual of Mental Disorders, fifth Edition (DSM-5). The final EIBDS consists of 25 items, all of which carry a 5-point range of responses, as follows: strongly disagree (0), disagree (1), unsure (3), agree (4), and strongly agree (5). The self-report measure of EIBDS was rated based on how the participants felt over the past month and if the items reflect participants’ emotional instability and behavioural difficulties. The total score ranges from 0-125, with higher scores reflecting greater emotional instability and behavioural difficulties. Some items were modified in order to retain the measured constructs and a few items were added. Based on this pilot test, a revised self-report measure had been administered to representative groups of youths.

### 2.2 Procedure

Community leaders from low-income apartments in the suburbs of Kuala Lumpur and Selangor were approached and a meeting organized where they agreed to help with the data collection. A master of list of household addresses was created which allowed for a sysmatic random sampling technique of household. Only one individual has been chosen to be parted of this data collection. A door-to-door distribution of survey questionnaires was undertaken. A decision of to distribute 600 questionnaire was made based on estimation of response rate of around 67 percent, which would yield a sample of 400 responses. For door-to-door distribution, 12 enumerators were engaged for a 2-month period to help the principle investigator to distribute the questionnaires door-to-door and collect them back. All participants were required to sign a consent form confirming their voluntary agreement to take part in this data collection, and authorizing the use of their anonymous data in future academic work or publications. In fact, there is no possibility for data to be traced back to a specific individual. For those youths below 18 years old, consent was obtained from their parents. The questionnaire was completed individually in the presence of enumerators and competent researchers during the data collection to clarify and answer any question from the youths. Face-to-face semi-structured interviews were applied for those who were illiterate or unable to fluently read or write.

Table 1 displays a translated version of the EIBDS. All items were translated into English version and reviewed by researchers who are fluent in both Malay and English languages. Table 2 shows internal consistency of the EIBDS using Cronbach's alpha, McDonald's ω, and Gutmann's λ6. Table 3 shows items reliability statistics, including mean, standard deviation, and item-rest correlation. The reliability of the EIBDS based on three types of reliability tests were good [4, 5, 6]. In this data, McDonald's ω was 0.938, the value of Cronbach's alpha was 0.936 and 0.954 for Gutmann's λ6. Discriminant validity and inner collinearity values were conducted using Partial Least Squares Structural Equation Modeling (PLS-SEM). Negative emotions were used to examine discriminant validity. The correlations provide evidence that the constructs were discriminated from each other (Table 4). The inner collinearity values were also performed to examine multicollinearity. Result showed there was no issue in multicollinearity in this data (Table 5).

**Table 1:** Translated version of the Emotional Instability and Behavioral Difficulties Scale (EIBDS)

Dimensions	The statement in Malay language (English language)
Sadness	B1. Saya mudah berasa pilu (I am easy to feel sorrow)
	B2. Saya mudah susah hati (I easily get upset)
	B7. Saya mempunyai banyak masalah (I have a lot of problems)
	B8. Hati saya mudah terluka (My heart is easily hurt).
	B9. Saya berasa sedih tanpa sebab (I feel sad for no reasons).
	B10. Saya mudah kecewa (I am easily disappointed)
	B11. Saya bermuram (I'm cheerless).
	B12. Saya mudah bimbang (I'm easily worried).
	B13. Saya cepat murung (I really depressed).
Anger	B14. Saya mudah marah (I easily get angry).
	B15. Saya panas baran (I'm hot-tempered).
	B19. Saya mudah meradang (I easily get furious).
	B20. Saya sering bertengkar dengan orang lain (I often argue with others).
Fear	B24. Saya mudah bimbang tentang apa jua perkara (I'm easily worried about anything).
	B26. Saya mudah takut (I'm easily afraid).
	B28. Saya mudah resah (I'm easily restless).
	B29. Saya mudah cemas (I'm easily anxious).
	B30. Saya berasa takut apabila memikirkan tentang sesuatu (I'm scared to think about something).
	B31. Saya mudah sentap (I'm easily jerky).
	B32. Saya cepat gugup (I'm easily nervous).
	B34. Saya mudah tersinggung (I'm easily offended).
Hyperactivity-inattention	C21. Saya mempunyai masalah dalam aktiviti/permainan yang melibatkan perancangan dan susun atur (lego/catur/dam/saidina/scrabble dll) (I have problems in activities/games involving planning and layout (lego/chess/dam/saidina/scrabble dll)
	C22. Saya mengelak melakukan tugasan yang memerlukan saya berfikir (I avoid doing the tasks that I need to think)
	C23. Saya selalu kehilangan barang penting untuk tugasan atau aktiviti (I always lose important stuff for assignment or activity).
	C24. Saya selalu lupa dalam melakukan aktiviti harian (I always forget about doing daily activities.)

**Table 2:** Reliability statistics of the EIBDS

Scale	McDonald's ω	Cronbach's α	Gutmann's λ6	Greatest lower bound
EIBDS	0.938	0.936	0.954	0.950

**Table 3:** Item reliability statistics

No	Mean	Standard Deviation	Item-test correlation	If item dropped		
				McDonald's ω	Cronbach's α	Gutmann's λ6
B1	2.386	0.972	0.638	0.934	0.932	0.952
B2	2.629	1.013	0.640	0.934	0.931	0.951
B7	2.507	0.997	0.563	0.935	0.933	0.953
B8	2.560	1.131	0.628	0.934	0.932	0.951
B9	2.025	1.021	0.624	0.935	0.932	0.952
B10	2.206	0.963	0.721	0.933	0.930	0.951
B11	1.993	0.941	0.701	0.933	0.931	0.951
B12	2.445	1.049	0.654	0.934	0.931	0.952
B13	1.983	0.967	0.691	0.933	0.931	0.951
B14	2.403	1.046	0.649	0.934	0.931	0.951
B15	2.090	1.065	0.559	0.936	0.933	0.952
B19	2.090	1.046	0.614	0.935	0.932	0.952
B20	1.858	0.852	0.479	0.937	0.934	0.953
B24	2.701	1.055	0.569	0.935	0.933	0.952
B26	2.396	1.021	0.626	0.935	0.932	0.951
B28	2.425	0.999	0.680	0.934	0.931	0.953
B29	2.333	0.949	0.695	0.934	0.931	0.951
B30	2.480	0.982	0.642	0.934	0.931	0.952
B31	2.465	1.059	0.646	0.934	0.931	0.951
B32	2.279	1.036	0.605	0.935	0.932	0.951
B34	2.443	1.037	0.655	0.934	0.931	0.952
C21	2.264	1.121	0.338	0.938	0.936	0.951
C22	2.353	1.069	0.405	0.938	0.935	0.951
C23	2.177	1.009	0.351	0.938	0.936	0.952
C24	2.400	1.014	0.284	0.939	0.936	0.952

**Table 4:** Analysis discriminant validity of the EIBDS using PLS-SEM

EIBDS constructs	1	2	3	4	5
1. Anger	0.83				
2. Negative emotions	0.47	0.8			
3. Fear	0.55	0.46	0.76		
4. Hyperactivity-inattention	0.34	0.38	0.33	0.77	
5. Sadness	0.61	0.44	0.73	0.25	0.78

**Table 5:** Inner multicollinearity values (VIF) using PLS-SEM

EIBDS constructs	Negative emotions
Anger	1.72
Fear	2.31
Hyperactivity-inattention	1.17
Sadness	2.5

[1]

## Declaration of Competing Interest

The authors declare that they have no known competing financial interests or personal relationships that could have appeared to influence the work reported in this paper.

## Appendix Supplementary materials

Image, application 1

## References

[1] Watson D.A, Clark L.A (1988). A Tellegen, Development and validation of brief measures of positive and negative affect: the PANAS scales. Journal of Personality and Social Psychology.

[2] Goodman R. (1997). The strengths and difficulties questionnaire: A research note. Journal of Child Psychology Psychiatry.

[3] Achenbach T.M. (1991). Manual for the Youth Self-Report.

[4] Bernardi R.A. (1994). Validating research results when Cronbach's alpha is below. 70: A methodological procedure, Educational and Psychological Measurement.

[5] Santos R.R.A., alpha Cronbach's (1999). A tool for assessing the reliability of scales. Journal of Extension.

[6] Vaske J.J., Beaman B., Carly C.S. (2017). Rethinking internal consistency in Cronbach's alpha. Leisure Sciences.

